# Possible Horizontal Gene Transfer of Novel Transposable Elements in *Anisakis simplex* between Hosts and Parasites

**DOI:** 10.17912/micropub.biology.001578

**Published:** 2025-09-02

**Authors:** Naoki Hoshino, Hiroki Kuroda

**Affiliations:** 1 Graduate School of Media and Governance, Keio University, Kanagawa, Japan; 2 Faculty of Environment and Information Studies, Keio University, Kanagawa, Japan

## Abstract

*Tc1* / *mariner* transposons found in salmoniform fish have been identified in both closely and distantly related fish species, suggesting that horizontal gene transfer may have occurred. However, the vectors of this process remain unknown. We identified two homologous sequences in the parasitic nematode *Anisakis simplex* , naming them *Tas1* ( T ransposable element of *A nisakis s implex* number 1 ) and *Tas2* . These elements encode *Tc1* / *mariner* transposases structurally similar to the active *Sleeping Beauty* transposase. Furthermore, *Tas1* / *2* were also identified in organisms that serve as hosts for *Anisakis* . These findings suggest that *Tas1* / *2* may have undergone horizontal gene transfer within host-parasite interactions.

**Figure 1. Tas1/2 form Tc1 / mariner transposases and undergo transposition and dissemination:**
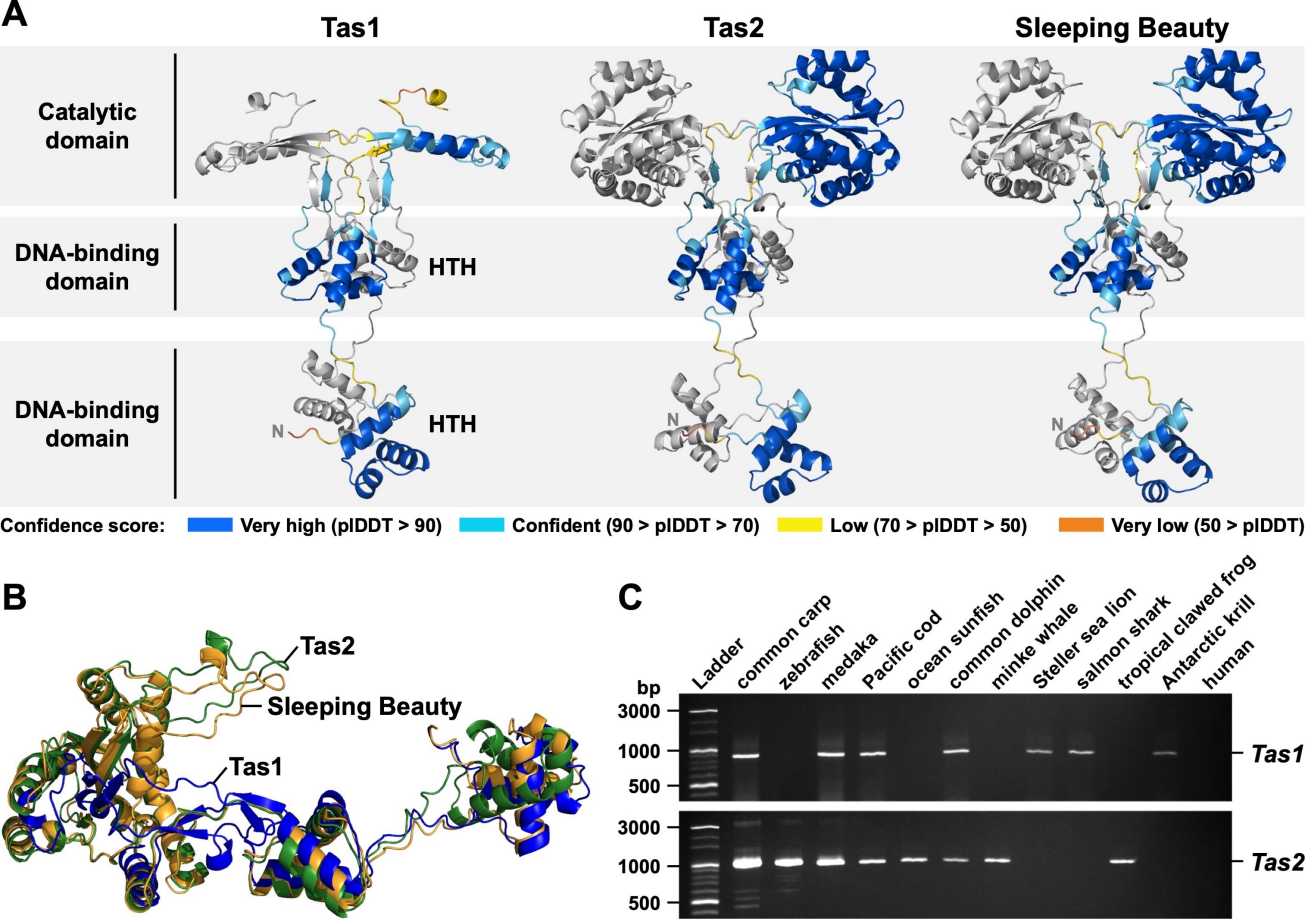
(A) Predicted three-dimensional structures of *Tas1* / *2* and *Sleeping Beauty* transposases. The catalytic domain and DNA-binding domain (HTH, h elix- t urn- h elix motif) are highlighted in light gray mesh. The colors of the structures indicate prediction confidence. (B) Superimposed three-dimensional structures of Tas1/2 and Sleeping Beauty. Tas1 is shown in blue, Tas2 in green, and Sleeping Beauty in orange. (C) PCR analysis targeting the internal sequences of *Tas1* / *2* . *Tas1* / *2* were detected in protostomes and deuterostomes that serve as hosts for *Anisakis* during its life cycle.

## Description

Transposable elements (TEs) are mobile genetic elements that can move within genomes. They were first discovered in maize by Barbara McClintock (McClintock, 1947; McClintock, 1950), and have since been identified in nearly all prokaryotic and eukaryotic organisms (Hickman et al., 2010; Kojima, 2020). Among them, the *Tc1* / *mariner* superfamily, a group of DNA (class II) TEs, is widely distributed across diverse taxa and represents one of the most widespread TE groups in eukaryotes (Brillet et al., 2007; Yuan and Wessler, 2011).

While TEs are typically inherited vertically from parent to offspring, they can also spread between species through horizontal gene transfer. In particular, *Tc1* / *mariner* have been frequently detected undergoing horizontal gene transfer and are recognized as a driving force in species diversity (Jia et al., 2022). These transposons have accumulated in high copy numbers in salmoniform fish (Goodier and Davidson, 1994; Krasnov et al., 2005) and are widely distributed among both closely and distantly related fish species, suggesting that transposition events were involved (de Boer et al., 2007). In eukaryotes, the horizontal gene transfer of TEs has been suggested to be mediated by viruses or parasites (Filée et al., 2015; Pace et al., 2008). However, empirical evidence and specific mediators remain largely unknown.

We conducted BLAST searches to investigate the distribution of *Tc1* / *mariner* across diverse taxa and found two sequences within the genome of the parasitic nematode *Anisakis simplex* (GenBank: UYRR01013216.1, UYRR01009494.1) that show high similarity to *Tc1* / *mariner* found in salmoniform fish. Further BLAST analyses using these sequences as queries revealed homologous elements across most genera within the order Salmoniformes, and high copy numbers were detected even on individual chromosomes. Moreover, PCR amplification was observed in *Salmo salar* , *Oncorhynchus tshawytscha* , *Salvelinus leucomaenis* , *Parahucho perryi* , and *Coregonus maraena* , all classified within the order Salmoniformes. Considering them to be TEs, we named these sequences *Tas1* ( T ransposable element of *A nisakis s implex* number 1 ) and *Tas2* .

*Anisakis* parasitizes various fish species, including salmoniform fish (Deardorff and Kent, 1989; Kuhn et al., 2013), and migrates across different hosts during its life cycle (Buchmann and Mehrdana, 2016; Klimpel et al., 2004). Therefore, *Tas1* and *Tas2* (collectively *Tas1* / *2* ) may have been acquired when *Anisakis* parasitized salmoniform fish, facilitating the horizontal gene transfer of *Tc1* / *mariner* . Additionally, as *Anisakis* moves through different hosts, it may serve as a vector for the further horizontal gene transfer of *Tas1* / *2* . This study investigates whether *Tas1* / *2* function as TEs and whether horizontal gene transfer has occurred between hosts and parasites to elucidate the mechanisms underlying horizontal gene transfer driven by TEs.

To determine whether *Tas1* / *2* encoded functional proteins, we predicted their three-dimensional structures using AlphaFold3. The predicted structures of *Tas1* / *2* closely resembled the transposase of *Sleeping Beauty* , an artificially reconstructed TE with transposition activity ( Figure 1A ). However, the GenBank-registered nucleotide sequence of *Tas1* was incomplete, and its predicted structure lacked some catalytic domains compared to standard *Tc1* / *mariner* sequences. We superimposed and aligned the 3D structures of Tas1/2 with Sleeping Beauty to compare their overall architecture. As expected, the catalytic and DNA-binding domains of Tas1/2 were well aligned with those of Sleeping Beauty, indicating structural similarity ( Figure 1B ). The root mean square deviation (RMSD) values, a measure of structural similarity, were 1.0 Å (partial) for *Tas1* and 4.3 Å (complete) for *Tas2* , further supporting their close resemblance.

*Tas1* / *2* may have transposed via transposases and contributed to horizontal gene transfer. To test this hypothesis, we performed PCR using primers targeting internal sequences of *Tas1* / *2* and identified amplification bands in intermediate hosts of *Anisakis* , including Pacific cod ( *Gadus macrocephalus* ) and ocean sunfish ( *Mola mola* ) (Santoro et al., 2020; Severin et al., 2020). We also detected bands in definitive hosts, such as common dolphins ( *Delphinus delphis* ), minke whales ( *Balaenoptera acutorostrata* ), and Steller sea lions ( *Eumetopias jubatus* ) (Berón-Vera et al., 2007; Nadler et al., 2005; Ugland et al., 2004). Furthermore, bands were observed in the salmon shark ( *Lamna ditropis* ), a predator of salmoniform fish and a definitive host of *Anisakis* (Gordeev and Sokolov, 2023), as well as in Antarctic krill ( *Euphausia superba* ), the primary intermediate host of *Anisakis* (Smith and Wootten, 1978) ( Figure 1C ).

Moreover, *Tas1* / *2* were identified in diverse vertebrates known to harbor various TEs, including common carp ( *Cyprinus carpio* ) and zebrafish ( *Danio rerio* ) (Chalopin et al., 2015; Xu et al., 2014), as well as in medaka ( *Oryzias latipes* ), which naturally carries the *Tol2* (Kawakami et al., 1998; Koga et al., 1996). Notably, *Tas2* were also found in the tropical clawed frog ( *Xenopus tropicalis* ), a species whose genome evolution and speciation have been linked to the inactivation of TEs (Session et al., 2016) ( Figure 1C ). These species did not show any PCR amplification bands when primers specific to the ITS1–2 region of the *Anisakis* genome were used, suggesting that *Anisakis* DNA contamination is unlikely and that *Tas1* / *2* are present in their genomes. These results indicate that *Tas1* / *2* have been widely spread and conserved across a broad range of organisms, including both protostomes and deuterostomes.

In conclusion, *Tas1* / *2* identified in *Anisakis* belong to the *Tc1* / *mariner* superfamily and are likely to encode active transposases. The presence of *Tas1* and *Tas2* in different host species suggests that they represent distinct TEs. Given the high abundance of *Tc1* / *mariner* in salmoniform fish and their extensive proliferation through transposition, *Anisakis* may have horizontally acquired *Tas1* / *2* when parasitizing salmoniform fish. Furthermore, the detection of *Tas1* / *2* in multiple *Anisakis* hosts indicates that *Anisakis* may have facilitated the horizontal gene transfer of these TEs between species as it cycles through various hosts. The transposases of *Tas1* / *2* exhibit a high degree of structural similarity to the *Sleeping Beauty* transposase, suggesting that they are likely autonomous transposons retaining transpositional activity. Therefore, it would be valuable for future studies to express *Tas1* / *2* both *in vivo* and *in vitro* and evaluate whether their transposases possess transposition activity. Naturally occurring transposons with active mobility are extremely rare, and those that are active are often utilized as vectors. If *Tas1* / *2* prove to be capable of transposition, they could serve as powerful new tools not only for genetic manipulation but also for large-scale genome engineering.

## Methods

**Protein Structure Prediction**

The amino acid sequences of *Tas1* / *2* were translated from nucleotide sequences using MEGA X (Kumar et al., 2018). The transposase of *Sleeping Beauty* , a representative *Tc1* / *mariner* (AOH76456.1), was retrieved from GenBank. Protein structures were predicted using AlphaFold3 (v3.0.0) (https://alphafoldserver.com) (Abramson et al., 2024). Structural analyses were conducted using PyMOL (version 3.1.3) (https://www.pymol.org) (DeLano, 2002).

**Structural Superimposition Analysis**

The PDB files of *Tas1* / *2* and *Sleeping Beauty* transposases were obtained from AlphaFold3 predictions. Structural alignment was conducted using the Dali server (http://ekhidna2.biocenter.helsinki.fi/dali/) to minimize the distances between corresponding Cα atoms in the transposases being compared (Holm, 2022). Structural similarity was evaluated by calculating the root mean square deviation (RMSD) based on the distances between all corresponding atomic coordinates (Cristobal et al., 2001).

**DNA extraction**

Muscle tissues from fish and amphibians were obtained by purchasing small tissue samples from local supermarkets. Approximately 10 mg of muscle tissue from each specimen was homogenized and completely digested in DNA extraction buffer (10 mM Tris-HCl [pH 8.0], 10 mM EDTA, 150 mM NaCl, 0.1% SDS) containing Proteinase K. Genomic DNA was subsequently purified by phenol-chloroform extraction, followed by isopropanol and ethanol precipitation. The extracted DNA was quantified and diluted with nuclease-free water to a final concentration of 10 ng/μL. For each sample, PCR was performed using primers specific to the ITS1–2 region of the *Anisakis* genome to confirm the absence of amplification products, thereby verifying the absence of *Anisakis* DNA contamination.

**PCR**

All primers were designed to have a melting temperature ( *Tm* ) between 50 and 55 °C. The following primers were used for PCR amplification. For *Tas1* , the forward ( *Tas1-fw* ) and reverse ( *Tas1-rv* ) primers were 5'-TGTATCACAATTCCAGTGGGTCAGAAG-3' and 5'-ATCCACATAATTTTCCTACCTCATGATGCC-3', respectively. The nested primers ( *Tas1-Nested-fw* and *Tas1-Nested-rv* ) were 5'-TTTACATACACTAAGTTGACTGTGCC-3' and 5'-ATCCACATAATTTTCCTACCTCATG-3'. For *Tas2* , the forward ( *Tas2-fw* ) and reverse ( *Tas2-rv* ) primers were 5'-GGCACCTGTTTGAACTTGTTATCAG-3' and 5'-GGTGGAAAATAAGTATTTGGTCAATAAC-3', respectively. The nested primers ( *Tas2-Nested-fw* and *Tas2-Nested-rv* ) were 5'-GACACCTGTCCACAACCTCAAAC-3' and 5'-CCTTTGTTGGCAATGACAGAGGTC-3'. The PCR reaction mixture (10 μL total volume) consisted of 1 μL extracted DNA, 5 μL EmeraldAmp MAX PCR Master Mix (#RR320A, TaKaRa), 0.1 μL each of forward and reverse primers (20 μM), and 3.8 μL nuclease-free water. PCR was performed using a LifeECO thermal cycler (#TC-96GHbC, Bioer Technology). For *Tas1* / *2* primers, the PCR conditions were as follows: 95 °C for 2 min; [95 °C for 30 s, 55 °C for 30 s, 72 °C for 1 min] × 35 cycles; final extension at 72 °C for 5 min. For *Tas1* / *2-Nested* primers, the annealing temperature was adjusted to 50 °C while maintaining the same conditions. Since TEs can exhibit various sizes due to mutations (Hayashi et al., 2022), non-specific amplification observed in *Tas1* / *2* PCR was resolved by performing nested PCR or touchdown PCR, using DNA extracted from the PCR product or gel bands as the template, to obtain a single band.
